# Evaluation of the Risk from Potentially Toxic Elements (PTEs) in Italy’s Most Consumed Processed Fish Products

**DOI:** 10.3390/foods13030456

**Published:** 2024-02-01

**Authors:** Maria Nobile, Giacomo Mosconi, Francesco Arioli, Luca Chiesa, Mariantonietta Peloso, Damiano Accurso, Elena Butovskaya, Giorgio Fedrizzi, Dalia Curci, Sara Panseri

**Affiliations:** 1 Department of Veterinary Medicine and Animal Science, University of Milan, Via dell’Università 6, 26900 Lodi, Italy; maria.nobile1@unimi.it (M.N.); giacomo.mosconi@unimi.it (G.M.); luca.chiesa@unimi.it (L.C.); dalia.curci@unimi.it (D.C.); sara.panseri@unimi.it (S.P.); 2 Istituto Zooprofilattico Sperimentale della Lombardia e dell’Emilia Romagna (IZSLER), Reparto Chimico degli Alimenti di Bologna, Via Fiorini 5, 40127 Bologna, Italy; m.peloso@izsler.it (M.P.); damiano.accurso@izsler.it (D.A.); giorgio.fedrizzi@izsler.it (G.F.); 3 Istituto Zooprofilattico Sperimentale della Lombardia e dell’Emilia Romagna (IZSLER), Reparto Chimica Alimenti e mangimi, Via Bianchi, 9, 25124 Brescia, Italy; elena.butovskaya@izsler.it

**Keywords:** potentially toxic elements, ICP-MS, PTEs, mass-spectrometry, fish preserves, fish

## Abstract

In a balanced diet, regular fish consumption provides positive outcomes for human health. On the other hand, the seafood supply chain faces a significant food safety risk due to the presence of potentially toxic elements (PTEs). In the present study, to assess the risk for Italian consumers, the concentrations of five PTEs, namely lead, chromium, cadmium, mercury, nickel, and aluminum, were determined in the three most consumed preserved fish in Italy: tuna (*Thunnus albacares, Katsuwonus pelamis*), mackerel (*Scomber scombrus*) and anchovy (*Engraulis encrasicolus*). Samples were collected from the national market, and the instrumental analysis was performed by inductively coupled plasma mass spectrometry (ICP-MS). The analyzed PTEs were found in all the species that were investigated. However, after considering the target hazard quotient (THQ) and the hazard index (HI), it was observed that the three fish preserves did not pose any risk of chronic toxicity for the average consumer, even at the highest concentrations detected. However, for significant consumers, mercury detected in tuna samples represented almost 90% of the tolerable weekly intake (TWI) reported by the European Food Safety Authority (EFSA), representing a matter of concern for consumers, particularly regarding developmental neurotoxicity, whose HI exceeded 111%. The acute toxicity of nickel was also considered for significant consumers at the highest concentration detected, and the margin of exposure (MOE) calculated was above 7000, much higher than the value of 30 indicated by EFSA. Due to the lack of data on non-professional carcinogenicity or human intake through foods with low cancer risk, this toxicity was not considered in the analysis of PTEs.

## 1. Introduction

Among the wealth of nutritional benefits that make fish and fish products essential for a healthy diet, fundamental are high-quality protein, essential nutrients, n-3 long-chain polyunsaturated fatty acids, vitamin D, calcium, iodine, selenium, and zinc [1]. The recommended human fish consumption in the diet is approximately 1–2 servings of seafood per week, increasing to 3–4 servings during pregnancy. This regular intake is associated with positive health outcomes, such as a lower risk of coronary heart disease (CHD) in adults and improved neurodevelopment in children [2]. Beyond its health benefits, fish consumption also holds cultural significance for many populations, where it serves as a primary food source [3].

Natural phenomena, such as weathering erosion, and anthropogenic inputs, such as urban wastewater discharges, can induce the pollution of the aquatic environment, leading to the accumulation of various toxic elements in the edible tissues of fish [4,5]. Specifically, through their skin, gills, and diet, fish absorb potentially toxic elements (PTEs) from the water [6]. The distribution of these elements in different fish tissues depends on factors such as the fish species, type of exposure, diet [7], and, significantly, the properties of the PTEs [8].

The acronym PTEs is used to define elements present in the environment due to natural emissions and anthropogenic activities [9], including cadmium (Cd), chromium (Cr), lead (Pb), mercury (Hg), nickel (Ni), and aluminum (Al). These elements are capable of bioaccumulation within the aquatic food chain, posing a risk to humans [10], who can be exposed to these elements by consuming contaminated fish and fish products. Especially in instances of long-term exposure, these elements can affect human health even at very low concentrations [11].

In particular, Cd, Pb, and Hg are included in the World Health Organization (WHO) list of the top 10 major health concern chemicals [12]. The European Food Safety Authority (EFSA) has expressed concern about their presence in foodstuffs [13,14]. Furthermore, there is growing interest in the occurrence of Al [15] and Ni [16], which are increasingly used as additives and packaging materials. Ni, in particular, has recently attracted more attention and requires further evaluation by the EFSA due to its potential health risks, especially for younger age groups, extending beyond individuals with nickel sensitivities [17]. Additionally, for Cr (VI), there is a need for data on its content, particularly in drinking water, to better define the risk assessment [18]. Detailed information about their toxicity is provided in the following paragraph.

The most harmful form of Hg is methylmercury [19], and its chronic exposure can lead to nervous system dysfunction, including tremors, irritability, memory problems, impaired vision, and hearing issues. Permanent nervous system dysfunction in children may result from maternal exposure. According to the Health-Based Guidance Value (HBGV) for methylmercury, a tolerable weekly intake (TWI) of 1.3 µg kg^−1^ b.w. per week has been established. As mentioned above, this value is specifically designated for assessing neurodevelopmental consequences resulting from prenatal exposure [14]. Regarding Cd, human exposure through the food chain can lead to reproductive toxicity [20], hepatic and hematological effects, neurological and digestive disorders, cancers, and dysfunction of the lungs, liver, and kidneys. The TWI for Cd is set at 2.5 µg kg^−1^ b.w. per week [13], with a focus on potential tubular damage. For Pb and its inorganic compounds, the toxic effects are associated with severe brain and kidney damage, as well as potential miscarriage. The benchmark dose lower confidence limit (BMDL) set for Pb considers neurodevelopmental toxicity, blood pressure, and kidney effects [21] with values specified at 0.50 (BMDL01), 1.5 (BMDL01), and 0.63 (BMDL10) µg kg^−1^ b.w. per day, respectively. Regarding Cr, chronic exposure can result in respiratory issues, including cough, asthma, and allergic reactions. Moreover, it exhibits different carcinogenic potential based on its oxidation state. Specifically, Cr (VI) is considered a carcinogen [22], but it is rare to find in food, where it undergoes reduction to Cr (III). The tolerable daily intake (TDI) for Cr (III) is set at 300 µg kg^−1^ b.w. per day, expressed for reproductive and developmental toxicity [18]. Al is classified as a human carcinogen [23], and its neurotoxicity has been linked to the development of Alzheimer’s disease [24]. The TWI for Al is set at 1.0 mg kg^−1^ b.w. per week, specifically addressing potential consequences on the developing nervous system [15]. Ni and its compounds are classified as carcinogens [23], with concerns over various health issues related to exposure through ingestion [25]. The TDI for nickel is 13 µg kg^−1^ b.w. per day, addressing reproductive and developmental toxicity, with the BMDL10 for nickel set at 1.3 µg kg^−1^ b.w. Meanwhile, the reference value for acute toxicity in sensitized individuals is the lowest observed adverse effect level (LOAEL) of 4.3 μg Ni kg^−1^ with a margin of exposure (MOE) of 30 due to individual sensitization-related response variability [17].

For these reasons, the seafood supply chain faces a significant food safety risk due to the presence of these substances in fishery products. “Calls for continuous collection of chemical contaminants occurrence data in food and feed” are periodically suggested by the EFSA, highlighting the importance of gathering data on the concentrations of these pollutants in food. Table 1 shows the current state of the topic, reporting some of the studies that have been conducted to acquire information about these contaminants and their spread in canned fish matrices. In particular, as we can see from Table 1, in the literature, there are several studies on a single type of fish or preserve in which the number of searched PTEs is variable (from 3 to 18 elements) and where the analytical protocol usually includes a sample digestion phase before analysis in IC OES or IC MS. No research has compared the accumulation of PTEs in the three main types of preserved fish (anchovy, tuna, and mackerel). Furthermore, each study offers a snapshot of the presence of PTEs within a limited geographical context, with the documented cases focusing on Turkey [26], the Arabian Gulf [27], the coasts of the Black Sea [28], Mediterranean [29], Sea Nigeria [30], and the central Adriatic Sea near the Jabuka Pit [31]. However, there is limited or no information available on risk characterization in these reported instances.

According to European regulations, maximum levels of PTEs in fish and fish products are set by Reg. 915/2023. However, it is important to note that maximum limits are not set for PTEs in canned and processed products [10]. Since the consumption of canned fish in 2022 accounted for a remarkable 24% of total fish consumption in Italy [32] and no maximum limits are set on these products, the present study aims to pursue the EFSA suggestion and collect up-to-date information on the concentration of these PTEs in the most widely consumed canned fish species in Italy. Therefore, we considered canned tuna (*Thunnus albacares, Katsuwonus pelamis*), anchovy (*Engraulis encrasicolus*), and mackerel (*Scomber scombrus*) as these are the most widely consumed preserved fish species in Italy, accounting for over the 97% of the total. Data were provided directly by ANCIT (Associazione Nazionale Corservieri Ittici e delle Tonnare—National Association of Fish Canners and Tuna-fishing Nets). Particularly, to simulate the behavior of an average Italian consumer, the samples were collected directly from the Italian market. A risk characterization was conducted to evaluate consumer risk related to PTE intake through canned fish consumption. Since the human exposure pathway to PTEs considered in the present work is related to diet, and considering that the carcinogen effects of the analyzed PTEs were often linked to different kinds of exposure, such as inhalation, the carcinogen index was not accounted for in the risk characterization. 

## 2. Materials and Methods

### 2.1. Chemicals and Reagents 

Fisher Scientific (Waltham, MA, USA) provided Hg, Ni, Cd, Cr, Pb, Al, and Yttrium (Y) (internal standard) (1000 mg L^−1^) while HNO_3_ (67–69 *v*/*v* % superpure) and HCl (34–37 *v*/*v* %) were provided by Carlo Erba (Cornaredo, Milan, Italy).

### 2.2. Sample Collection 

A total of 95 samples of canned fish were collected from Italian markets, including 45 cans of tuna, 28 jars of anchovies, and 22 cans of mackerel. Samples were purchased directly from the Italian market without considering the different geographical origins of the fish.

### 2.3. Analytical Protocol 

Polypropylene tubes (Digi-Tubes SCP Science (QuantAnalitica SRL, Osnago, Italy)) were used to weigh 5 g of fish muscle for a wet mineralization process. To this, 10 mL of concentrated nitric acid was added and heated overnight at 75 ± 10 °C. Digestion was conducted in closed propylene tubes in a temperature-controlled mineralizer (Digi-Prep SCP-Science, (QuantAnalitica SRL, Osnago, Italy)). After mineralization, the solution was made up to volume with high-purity deionized water (Evoqua WaterTechnologies, Pittsburgh, PA, USA), filtered through paper filters, and 1 mL of the obtained solution was diluted to 10 mL with a dilution solution (an aqueous solution of 2% (*v*/*v*) nitric acid and 0.5% (*v*/*v*) hydrochloric acid). Analysis was conducted via inductively coupled plasma-mass spectrometry (ICPMS 7700 Series Agilent Technologies Inc. Santa Clara, CA, USA) using an ASX-500 CETAC autosampler (Cetac Technologies, Omaha, NE, USA). Operating parameters included an RF power of 1.55 kW, a plasma gas (argon-Ar) flow of 15 L min^−1^, a carrier gas (argon-Ar) flow of 1.01 mL min^−1^, a cell gas flow (helium-He) for the “He” mode of 5 mL min^−1^. The isotopes (*m*/*z*) monitored were Hg202 and Pb208 in “He” mode. The concentrations were calculated using solvent calibration curves and calibration standards provided by Agilent Technologies Inc. (Santa Clara, CA, USA). A diluted solution was prepared for the reference materials. For each series of analyses, a calibration curve from 0.01 to 100 ng mL^−1^ was analyzed, and the correlation coefficient was equal to or greater than 0.999 for each element subjected to analysis. The accuracy of the method was determined by analyzing the certified reference materials (Community Bureau of Reference-BCR-185R Bovine Liver and ERM-BB422 Fish Muscle (Merck, Darmstadt, Germany)) in each lot. The concentration values of the reference materials were within the confidence interval indicated by the BCR—and are reported in Table S2. For each series of analyses, a blank sample was mineralized and treated as described above. As it was not always possible to find samples free of analytes of interest, each blank matrix’s signal (measured in CPS—counts per second) was subtracted from the corresponding fortified sample during validation prior to concentration determination. At the beginning of each measurement cycle, a tuning operation was performed with a mixture of several elements to verify the accuracy of the identification of the *m*/*z* ratio values and the accuracy of the instrument. The average content of the elements was expressed in mg kg^−1^. The limit of detection (LOD) was set at 3 µg kg^−1^ and the limit of quantification (LOQ) at 5 µg kg^−1^ for all matrices.

### 2.4. Method Validation

The method exhibited good linearity for cadmium, lead, chromium, mercury, nickel, and aluminum concentrations ranging from 0.003 to 2 mg kg^−1^. The correlation coefficient obtained from the construction of the corresponding straight lines was greater than 0.998 for each metal subjected to analysis. The method is selective, as matrix blanks (fish muscle, crustaceans, mollusks, and cephalopods) were analyzed for each validation session, and no significant interferences were detected. The percentage of recovery relative to each concentration was assessed by analyzing blank samples fortified with cadmium, lead, chromium, mercury, and nickel at 5, 200, and 500 µg kg^−1^ and aluminum at 50, 500, and 1000 µg kg^−1^. All subsequent evaluations, including the current one, were conducted using counts obtained from the instrument without evaluating the corrections made by the internal standard. Each determination was conducted on 3 separate days in 6 replicates for each day according to the procedure outlined above. Repeatability was assessed by analyzing 6 replicate samples fortified with cadmium, lead, chromium, mercury, and nickel at 5, 200, and 500 µg kg^−1^ and with aluminum at 50, 500, and 1000 µg kg^−1^. Each determination was conducted according to the above procedure. Intra-laboratory reproducibility was assessed by analyzing 18 replicates on 3 separate days (6 replicates per day) of the samples fortified with cadmium, lead, chromium, mercury, and nickel at 200, 500, and 1500 µg kg^−1^ and in 24 replicates on 3 separate days (6 replicates per day) the samples fortified with aluminum at 50, 500 and 1000 µg kg^−1^. The detection limit evaluated in the statistical data processing is 3 µg kg^−1^, and the limit of quantification assessed is 5 µg kg^−1^. The verification of the LOD and LOQ for metal analysis was not properly conducted according to Regulation 333/2007 and subsequent amendments but was assessed in a more restrictive manner. The laboratory qualitatively/quantitatively assessed the LOD and LOQ by inserting them into matrix calibration straight lines (3 and 5 µg kg^−1^). It verified that the point of interest of the curve (1 µg kg^−1^) was at least 5 times that of the blank, considering the number of CPS during validation and for each analytical batch. Furthermore, as it was not always possible to find samples free of analytes of interest, each blank matrix at validation was subtracted (in CPS) from the respective fortified sample before the concentration determination.

### 2.5. Statistical Analysis

Statistical analyses were conducted using Graphpad Instat 3 software, version 3.10 (Graphpad Instat Software, San Diego, CA, USA). As the Kolmogorov–Smirnov test for normality stated a Gaussian distribution only for Pb in anchovies and Cd in mackerels, the Kruskal–Wallis test (non-parametric ANOVA) was used for the comparison of populations, and Dunn’s multiple comparisons test as a post-test, considering the null-hypothesis when P was lower than 0.05. 

### 2.6. Risk Characterization Protocol

Consumption data for preserved fish were retrieved from Ismea’s report [32] (Ismea, Istituto di Servizi per il Mercato Agricolo Alimentare—Agricultural Food Market Services Institute). Furthermore, to obtain information on the g die^−1^ per capita of different fish consumed, we used the percentage of consumption of the different products provided by ANCIT and the Italian population over 2 years old. The decision to exclude the population under 2 years of age was motivated by the WHO’s recommendation that until this age, there is a gradual approach to a diet common to that of the rest of the family [33]. We also excluded vegans and vegetarians. However, we could not find data from official agencies. Regarding their prevalence in the Italian population, the only available information is derived from a market survey commissioned by a major insurance company, focusing on aspects related to health and healthcare. According to the report, 8 percent of Italians aged 18 and above fall into this consumer category [34].

The risk characterization for human health was performed using several parameters. Minimum and first to fourth-quartile concentrations were considered for each element and each species. The parameters used were HBGV, estimated daily intake (EDI), targeted hazard quotient (THQ), and hazard index (HI). To adopt a more conservative approach regarding Hg, all of its concentration was considered as methylmercury, the most toxic form of mercury. 

As reported by the EFSA, “The Health-Based Guidance Values (HBGV) is a science-based recommendation for the maximum (oral) exposure to a substance that is not expected to result in an appreciable health risk, taking into account current safety data, uncertainties in these data, and the likely duration of consumption” [35]. For the analyzed PTEs, the considered HBGV were reported in “Section 1”. 

The estimated daily intake (EDI) of the PTEs was calculated as follows:

EDI = C × DC/BW,(1)

where C is the median concentration, DC is the daily fish consumption per capita in Italy, and BW is the assumed consumer body weight, set at 70 kg. We also considered the estimated fish consumption of the 95th percentile consumers, using data from Leclercq et al., 2009 [36]. This involved determining a proportion factor of 3.7 by dividing the 95th percentile consumption value of preserved fish and seafood by the median consumption value. 

The target hazard quotient (THQ), which is the ratio between the exposure and the EFSA Health-Based Guidance Value (HBGV), reported in the introduction for each element and each end-point, was subsequently recalculated daily as follows:

THQ = EDI/HBGV,(2)

For a very conservative approach, we also used the highest concentration found among the samples.

The hazard index (HI) [1] for similar toxic effects of the different PTEs was therefore calculated as follows:

(3) $$\mathrm{HI}=\sum_{i=8}^{n}THQ,$$

## 3. Results and Discussion

### 3.1. Validation Parameters

According to the validation performance of the method, detailed information is reported in Table 2. 

### 3.2. Occurrence of PTEs in Canned Fish Products

Raw data of PTE concentrations are reported in Table S1, while data expressed as median and quartiles are shown in Table 3. Results showed that Pb and Cd median and maximum value concentrations were higher in anchovy than in mackerel and tuna. Cr was higher in mackerel due to a higher detection prevalence, even if the highest value is in anchovy. As expected, Hg was higher in tuna than other species. This is an interesting finding as the species of tuna used for canning are smaller than those usually used for fresh consumption and so less subjected to bioaccumulation and biomagnification. Tuna, therefore, remains a fish to be particularly careful about, especially for those population groups at risk, such as infants and pregnant women. Anchovy showed the higher media concentration of Ni, while tuna showed the highest. Al concentration is unexpectedly higher in anchovy preserved in glass jars, unlike mackerel and tuna in aluminum cans. This leads to the assumption that the presence of aluminum is not due to a transfer from the packaging but rather to the uptake by the fish from their environment [6]. 

The findings of the current study, when compared with those reported by Nobile et al. 2023 [10], show that in the case of anchovy, lower concentrations were observed for all analytes, except for Al, which exhibited the highest concentration detected, and Ni, which showed a median concentration disparity. Meanwhile, for the prevalence, we observed higher results for Hg (100% vs. 71%) and lower results for Cr (50% vs. 100%). Considering the Cd, Pb, and Hg results of Ulusoy et al. 2023 [4] in canned tuna, we observed slightly higher concentrations only for Hg. Regarding mackerel, our results are lower than those found by Perugini et al., 2013 [31], which found mean concentrations of 0.03, 0.36, and 0.05 mg kg^−1^ for Cd, Hg, and Pb, respectively.

### 3.3. Risk Characterization 

As of the start of 2022, the Italian population, as reported by the National Institute of Statistics (ISTAT), stood at 54,149,751 individuals. This count excludes infants aged 0 to 2 years and individuals aged over 18 who follow vegan or vegetarian diets, as previously specified. The total volume of preserved seafood was 139.8 tons, with tuna accounting for 87%, mackerel for 7%, and anchovies for 3.6%. The consumption calculated from these values was 6.15, 0.25, and 0.50 g die^−1^ per capita for tuna, anchovy, and mackerel, respectively. Taking these considerations into account, an extensive risk characterization was conducted, representing the strength of this work.

The calculated THQ and HI are reported in Table 4, Table 5 and Table 6, respectively. 

The HI calculated for reproduction development, ascribed to the presence of Ni and Cr, remained below 0.1% for all the species considered, except anchovy at its highest detected concentration among large consumer groups. This exception does not raise concerns for consumers. The same considerations can be applied to the effects on blood pressure and kidney toxicity, with HI values exceeding 1% only for tuna, reaching a maximum of 4% and 5%, respectively, for average and large consumers, as shown in Table 5 and Table 6. The most worrying results, however, were found in the effects on neuronal development due to the effects of lead and mercury present in tuna. This results in an HI of 30% for average consumers at the maximum detected concentration. In the case of heavy consumers, the HI escalates to 111%, considering once again the maximum detected concentration. These results are largely due to the concentration of mercury present in the tuna samples, with a median of 0.093 mg kg^−1^, while the maximum value reaches 0.63 mg kg^−1^. Regarding Cr, the different toxicities of Cr(III), which exhibits reproductive and developmental toxicity, and Cr(VI), classified as a carcinogen, were previously considered for two main reasons [37]. Firstly, the expectation is that ingested Cr(VI) is reduced in the stomach’s acidic environment, minimizing the health risks [38]. Secondly, for the same reason, Cr(III) is the form found in food [39]. Inhalation or dermal contact of Cr(VI) is much more problematic than oral ingestion [37]. Therefore, the current study categorizes all detected Cr as Cr(III), omitting consideration of the carcinogenic effects associated with Cr(IV). 

In individuals with sensitivities, Ni can also cause the occurrence of systemic dermatitis following acute exposition. The EFSA [17] set the reference value at 4.3µg kg^–1^, with a margin of exposure (MOE) of 30. Specifically, we used the individual portion consumption data established by the Food and Drug Administration (FDA) [40], which are 15 g, 85 g, and 85 g for anchovy, mackerel, and tuna, respectively. The highest intake value, 0.00061 µg kg^–1^, was found for tuna, regarding the consumption patterns of heavy consumers and the highest concentration detected. By comparing the value of HBGV with the actual intake value, we obtained a value of 7049, over 230 times higher than the indicated MOE of 30. Nickel, therefore, does not seem to constitute a concern with regard to possible adverse reactions due to its acute intake.

## 4. Conclusions

Although the analyzed PTEs were found in all the investigated species, the risk characterization, which represents the strength of this work, showed that the concentration detected does not represent a concern for Italian consumers. However, the values of THQ and HI obtained for heavy consumers, especially those associated with substantial tuna consumption, suggest that limiting the intake of tuna preserves to 1–2 servings per week is advisable. Moreover, considering the acute toxicity of Ni, the comparison between the obtained values and the HBGV suggested by the EFSA indicates that the intake of Ni through a single portion of tuna, anchovy, and mackerel does not pose a risk for adverse reactions in sensitive people. Nevertheless, given the widespread presence of PTEs in food matrices, it is advisable to consider a wide variety of foods and beverages consumed daily to perform a comprehensive analysis of the effect of PTEs on human health. 

## Figures and Tables

**Table 1:** Literature overview regarding detection of PTEs in different fish products.

Reference	Analytes	Matrix	Extraction Technique	Instrumental Analysis	Limits of the Method (ng g^−1^)	Application Range Concentration (ng g^−1^)
Anchovy						
[10]	Hg, Cd, Pb, Cr, As, Sn, Al, Ni	Salted and canned anchovy	Acid digestion with HNO_3_, H_2_O_2_, H_2_O.	ICP OES	LOQ = 1.2–12 LOD = 0.40–3.6	Hg, Cd, Pb, Cr, iAs, Sn, Al and Ni = 90.00–4940
[26]	Fe, Zn, Cu, Cd, Sn, Hg and Pb	Canned anchovy and canned rainbow trout	Digestion with HNO_3_ and H_2_O_2_. Microwave and washing.	ICP MS	/	1.0–5.1 × 10^4^
[27]	As, Cd, Co, Cr, Cu, Mn, Mo, Ni, P, Pb, V, Zn, Ca, K, Na, Mg, S and Sr	Anchovy	Digestion with nitric acid (65%).	ICP OES	LOD = 1.0–4.9 × 10^5^	40–75 × 10^5^
[28]	Al, Zn, Mn, Co, Cr, Cu, Fe, Ni, Cd, Pb, Se, As and Hg	Anchovy	Homogenization and drying of the samples followed by digestion with nitric acid and hydrochloric acid. Dilution and filtration.	ICP MS	LOD = 0.10–29	3.0–14 × 10^2^
**Tuna**						
[4]	Cd, Pb, Hg, As	Canned tuna	Digestion with nitric acid (65%) and hydrogen peroxide (30%). Microwave.	ICP MS	LOD = 0.025–0.18 LOQ = 0.045–0.54	0.01–2.55
[29]	Cd, Pb and Fe	Wild and farmed Atlantic bluefin tuna	Homogenization and sample freezing. Microwave assisted digestion with HNO_3_ and H_2_O_2_.	GFAAS	/	0.7–31,000
**Mackerel**						
[30]	Pb, Hg, Cr, As, and Cd	Mackerel	Homogenization and digestion.	AAS	/	ND—4000
[31]	As, Cd, Hg, Pb, Cu, Zn and Se	Different species including Mackerel	Homogenization and sample freezing. Microwave assisted digestion with HNO_3_, H_2_O_2_ and HF.	ICP OES	LOD = 0.0020–0.10	30–360

ICP OES—inductively coupled plasma optical emission spectroscopy; ICP MS—inductively coupled plasma mass spectrometry; GFAAS—graphite furnace atomic absorption spectrometry; AAS—atomic absorption spectrometry; LOD—limit of detection; LOQ—limit of quantification; ND—not detected.

**Table 2:** Method validation parameters were obtained by spiking blank samples at the reported concentration (RSD_r_: repeatability; RSD_R_: within-laboratory reproducibility; Horrat r, Horrat R, and mean recovery %).

Analyte	Conc. (µg kg^−1^)	Uncertainty ^a^ % (Û)	RSD_r_	Horrat r ^b^	RSD_R_	Horrat R ^b^	Mean Recovery %
Cd	5	21.1	16.1	0.460	15.2	0.435	
	200		6.3	0.306	7.4	0.361	86.6
	500		6.3	0.348	8.5	0.474	
Pb	5	21.4	6.1	0.174	5.95	0.168	81.0
	200		6.5	0.305	8.8	0.408	
	500		7.1	0.378	8.5	0.455	
Cr	5	20.6	8.9	0.256	13.6	0.389	
	200		8.30	0.405	11.4	0.552	85.4
	500		5.9	0.331	10.3	0.570	
Hg	5	20.4	15.5	0.684	19.2	0.535	
	200		7.6	0.377	9.2	0.450	91.1
	500		4.3	0.243	7.6	0.428	
Ni	5	22.0	12.9	0.586	12.3	0.558	
	200		2.4	0.115	9.4	0.439	81.5
	500		2.3	0.125	9.3	0.498	
Al	50	26.2	3.47	0.378	5.94	0.651	
	500		1.38	0.213	3.38	0.522	87.6
	1000		2.80	0.479	3.73	0.641	

^a^ The expanded uncertainty of the method (U_exp_ at 95% confidence level and k = 2) was calculated using a bottom-up approach on six replicates. ^b^ RSD_r_ and RSD_R_ observed values were divided by the respective values calculated from the Horwitz equation.

**Table 3:** Minimum, 1st quartile, median, 3rd quartile, maximum values, and above LOQ percentage (>LOQ %) of potentially toxic elements in the samples analyzed. Concentration values expressed in mg Kg^−1^. Differences in concentrations of the same elements among the different species are denoted by asterisks, as explained in the footnote. The highest median value of each potentially toxic element is highlighted in bold.

	Pb	Cd	Cr	Hg	Ni	Al
Tuna n = 45						
Minimum	0.00	0.00	0.031	0.00	0.170	0.018
1st quartile	0.010	0.0080	0.00	0.060	0.00	0.11
Median	0.013	0.013	0.0090	0.093	0.00	0.13
3rd quartile	0.017	0.020	0.013	0.14	0.0060	0.19
Maximum	0.025	0.065	0.045	0.63	0.028	0.63
>LOQ %	89	96	64	100	47	100
Different from	anchovy ***	anchovy ***	mackerel **	anchovy *** mackerel **	anchovy *** mackerel ***	anchovy *** mackerel ***
**Anchovy n = 28**						
Minimum	0.012	0.025	0.00	0.029	0.018	0.094
1st quartile	0.040	0.036	0.00	0.034	0.032	0.62
Median	0.047	0.19	0.0030	0.039	0.21	1.45
3rd quartile	0.053	0.22	0.0080	0.069	0.24	2.00
Maximum	0.082	0.23	0.14	0.21	0.33	6.60
>LOQ %	100	100	50	100	100	100
Different from	tuna *** mackerel ***	tuna *** mackerel ***	mackerel ***	tuna ***	tuna *** mackerel ***	tuna *** mackerel ***
**Mackerel n = 22**						
Minimum	0.0090	0.00	0.00	0.00	0.00	0.00
1st quartile	0.010	0.0070	0.011	0.021	0.0080	0.00
Median	0.012	0.013	0.014	0.027	0.013	0.00
3rd quartile	0.014	0.018	0.024	0.042	0.016	0.00
Maximum	0.022	0.035	0.083	0.083	0.054	0.41
>LOQ %	100	86	95	95	95	9
Different from	anchovy ***	anchovy ***	tuna ** anchovy ***	anchovy ***	tuna *** anchovy ***	tuna *** anchovy ***

***: *p* < 0.001; **: *p* < 0.01.

**Table 4:** Target hazard quotients (THQ) result from the estimated daily intake (EDI) of average consumers to the potentially toxic elements (PTEs) through the consumption of the studied preserved fish species at the highest concentrations. The Health-Based Guidance Values (HBGV), established by the EFSA, when set on a weekly basis were recalculated and expressed on a daily basis for assessment. In the case of Cr, only Cr(III) is considered.

HBGV	PTE	Tuna	Anchovy	Mackerel	THQ Tuna–Anchovy–Mackerel (HBGVs µg kg^−1^ day^−1^)				
		EDI µg kg^−1^			Skin	Reproduction Development	Developmental Neurotoxicity	Blood Pressure	Kidney
BMDL_10_	Pb	0.0022	0.00030	0.00016			0.0044–0.00060–0.00031 (0.5)	0.0015–0.00020–0.00010 (0.15)	0.0035–0.00047–0.00025 (0.63)
TWI	Cd	0.0057	0.00085	0.00025					0.016–0.0024–0.00069 (0.36)
TDI	Cr	0.0040	0.00050	0.00059		0.000013–0.0000020–0.0000020 (300)			
TWI	Hg	0.055	0.00078	0.00059			0.30–0.0042–0.0032 (0.19)		
TDI	Ni	0.0025	0.0012	0.00038		0.00019–0.000091–0.000029 (13)			
TWI	Al	0.055	0.024	0.0029			0.00039–0.00017–0.000020 (143)		

**Table 5:** Hazard indexes (HI) for the estimated daily exposure to the studied elements through tuna, anchovy, and mackerel at the highest concentration detected by average consumers.

	Reproduction Development	Developmental Neurotoxicity	Blood Pressure	Kidney
	Ni + Cr	Pb + Hg	Pb	Pb + Cd
Tuna	0.00020	0.30	0.015	0.019
Anchovy	0.000093	0.0048	0.0020	0.0028
Mackerel	0.000031	0.0035	0.0010	0.00094

**Table 6:** Hazard indexes (HI) for the estimated daily exposure to the studied elements through tuna, anchovy, and mackerel at the median, 3rd quartile, and highest concentration detected among the 95th percentile of consumers.

		Reproduction Development	Developmental Neurotoxicity	Blood Pressure	Kidney
		Ni + Cr	Pb + Hg	Pb	Pb + Cd
Tuna	median	0.000010	0.17	0.028	0.019
	3rd quart	0.00016	0.25	0.037	0.027
	max	0.00075	1.11	0.054	0.072
Anchovy	median	0.00022	0.0041	0.0042	0.0081
	3rd quart	0.00024	0.0064	0.0048	0.0092
	max	0.00034	0.018	0.0022	0.011
Mackerel	median	0.000027	0.0044	0.0021	0.0014
	3rd quart	0.000034	0.0066	0.0024	0.0019
	max	0.00012	0.013	0.0038	0.0035

## Data Availability

Data is contained within the article or supplementary material.

## Supplementary Materials

The following supporting information can be downloaded at https://www.mdpi.com/article/10.3390/foods13030456/s1. Table S1: PTE concentrations expressed in mg kg^−1^. Table S2: Average values of metals of interest for certified materials checked during fish products analysis.
