# Supplementary material for: Evaluation of the Risk from Potentially Toxic Elements (PTEs) in Italy’s Most Consumed Processed Fish Products
Source: Foods. 2024 Feb 1;13(3):456. doi: 10.3390/foods13030456 (PMC10855315; doi:10.3390/foods13030456)
Supplement: Supplementary file 1 [file foods-13-00456-s001.zip › foods-2807774-supplementary.pdf]

Table S1: PTE concentrations expressed in mg Kg^{-1}

A: Canned tuna

Pb	Cd	Cr	Hg	Ni	Al
0.020	0.016	0.013	0.14	0.0090	0.24
0.011	0.0070	0.0090	0.093	<LOD	0.036
0.017	0.0060	0.010	0.088	<LOD	0.15
0.017	0.0090	0.014	0.11	0.0070	0.15
0.016	0.010	0.0090	0.17	<LOD	0.13
0.018	<LOD	0.011	0.089	0.0050	0.19
0.014	0.013	0.014	0.071	0.0060	0.13
0.018	0.0060	0.014	0.10	0.0060	0.24
0.011	0.0050	0.045	0.14	<LOD	0.13
0.013	<LOD	<LOD	0.38	0.0060	0.16
<LOD	0.0080	<LOD	0.17	<LOD	0.038
0.013	0.065	0.0050	0.073	0.0080	0.12
0.011	0.046	<LOD	0.037	<LOD	0.13
0.011	0.016	<LOD	0.058	<LOD	0.079
0.010	0.023	<LOD	0.058	<LOD	0.089
0.010	0.021	<LOD	0.031	<LOD	0.13
0.014	0.026	<LOD	0.11	0.0050	0.092
0.011	0.017	<LOD	0.096	<LOD	0.095
<LOD	0.0090	0.030	0.065	0.0090	0.082
0.016	0.022	0.013	0.048	<LOD	0.25
0.016	0.015	0.0090	0.087	0.010	0.21
0.015	0.020	0.011	0.091	0.0060	0.018
0.012	0.013	0.0090	0.095	<LOD	0.13
0.018	0.025	0.0090	0.12	0.0070	0.21
0.017	0.010	0.013	0.060	0.0050	0.11
0.016	0.014	0.011	0.093	0.017	0.054
0.016	0.015	0.010	0.11	0.0060	0.11
0.018	0.027	0.013	0.058	0.0050	0.19
0.010	0.0060	<LOD	0.45	<LOD	0.63
<LOD	0.0090	0.015	0.053	0.018	0.020
0.0090	0.016	<LOD	0.042	<LOD	0.15
<LOD	0.013	<LOD	0.63	<LOD	0.14
<LOD	0.012	<LOD	0.040	<LOD	0.23
0.013	0.012	<LOD	0.073	<LOD	0.12
0.012	0.032	<LOD	0.17	<LOD	0.11
0.011	0.013	0.007	0.21	<LOD	0.18

0.01	0.036	0.017	0.43	<LOD	0.37
0.0090	0.0080	0.011	0.19	<LOD	0.34
0.017	0.057	0.010	0.039	0.028	0.25
0.018	0.016	<LOD	0.13	<LOD	0.12
0.016	0.006	<LOD	0.040	<LOD	0.088
0.025	0.0060	0.013	0.25	0.0060	0.18
0.016	0.0080	0.011	0.061	0.0050	0.21
0.017	0.0080	0.012	0.12	0.0060	0.19
0.010	0.0070	0.011	0.10	<LOD	0.12

B: Jarred anchovies

**Pb**	**Cd**	**Cr**	**Hg**	**Ni**	**Al**
0.039	0.036	0.014	0.16	0.030	0.89
0.058	0.090	0.0070	0.061	0.063	1.2
0.031	0.032	<LOD	0.048	0.018	0.58
0.035	0.041	<LOD	0.074	0.032	0.80
0.035	0.036	0.024	0.21	0.051	0.17
0.044	0.029	0.0060	0.085	0.038	0.62
0.039	0.056	0.014	0.045	0.018	0.24
0.012	0.027	<LOD	0.075	0.022	0.63
0.042	0.089	0.0080	0.055	0.037	0.22
0.041	0.12	<LOD	0.067	0.064	0.094
0.054	0.027	0.0080	0.067	0.025	1.0
0.050	0.025	0.0070	0.075	0.023	0.39
0.054	0.030	0.015	0.078	0.028	0.95
0.051	0.23	<LOD	0.038	0.21	2.2
0.046	0.20	0.14	0.033	0.23	2.4
0.082	0.20	<LOD	0.034	0.27	2.5
0.053	0.23	<LOD	0.037	0.24	1.5
0.047	0.19	<LOD	0.031	0.22	2.0
0.038	0.20	0.013	0.029	0.23	1.5
0.055	0.19	0.0070	0.031	0.25	6.6
0.063	0.21	0.0070	0.032	0.33	2.4
0.045	0.21	<LOD	0.032	0.23	1.7
0.053	0.23	0.0070	0.034	0.22	1.4
0.052	0.23	<LOD	0.039	0.24	2.1
0.047	0.23	<LOD	0.034	0.24	2.0
0.046	0.23	<LOD	0.035	0.21	1.8
0.050	0.22	<LOD	0.034	0.24	2.0

0.059	0.20	<LOD	0.037	0.24	1.9

B: Canned mackerel

Pb	Cd	Cr	Hg	Ni	Al
0.011	<LOD	0.083	<LOD	0.024	<LOD
0.021	0.0070	<LOD	0.071	0.022	<LOD
0.021	0.018	0.0080	0.029	0.036	<LOD
0.010	<LOD	0.043	0.042	0.013	<LOD
0.010	0.010	0.043	0.053	0.013	<LOD
0.0090	0.0060	0.017	0.083	0.011	<LOD
0.013	0.018	0.031	0.026	0.019	<LOD
0.010	0.0070	0.024	0.040	0.012	<LOD
0.011	0.035	0.054	0.053	0.054	<LOD
0.022	0.014	0.020	0.018	0.013	<LOD
0.013	<LOD	0.024	0.041	0.015	<LOD
0.011	0.0050	0.014	0.078	0.014	<LOD
0.012	0.019	0.016	0.030	0.016	<LOD
0.011	0.010	0.011	0.027	0.013	<LOD
0.012	0.014	0.011	0.020	0.0070	<LOD
0.016	0.021	0.012	0.020	0.0090	<LOD
0.014	0.014	0.0080	0.026	0.0070	0,41
0.014	0.017	0.013	0.019	0.010	0,032
0.010	0.012	0.012	0.027	0.0060	<LOD
0.012	0.014	0.012	0.022	0.0080	<LOD
0.010	0.021	0.009	0.023	<LOD	<LOD
0.013	0.011	0.008	0.021	0.0050	<LOD

Table S2: Average values of metals of interest for certified materials checked during fish products analysis

Sample	Elements	Found (mg kg^{-1})	Certified (mg kg^{-1})
**BCR 185R**	**Cd**	0.498 ±0.171	-0.544 ±0.017
	**Pb**	0.169 ±0.061	0.172 ±0.009
**ERM-BB422**	**Hg**	0.570 ±0.185	0.610 ±0.030
